# SnapKi—An Inertial Easy-to-Adapt Wearable Textile Device for Movement Quantification of Neurological Patients

**DOI:** 10.3390/s20143875

**Published:** 2020-07-11

**Authors:** Ana Oliveira, Duarte Dias, Elodie Múrias Lopes, Maria do Carmo Vilas-Boas, João Paulo Silva Cunha

**Affiliations:** 1 Biomedical Research and INnovation (BRAIN), Centre for Biomedical Engineering Research (C-BER), INESC Technology and Science, 4200-465 Porto, Portugal; ana.e.oliveira@inesctec.pt (A.O.); elodie.murias2@gmail.com (E.M.L.); carmo.vilas.boas@inesctec.pt (M.d.C.V.-B.); jpcunha@inesctec.pt (J.P.S.C.); 2 Faculty of Engineering, University of Porto, 4200-465 Porto, Portugal; 3 Centro Hospitalar do Porto, Hospital Santo António, Unidade Corino de Andrade, E.P.E., 4099-001 Porto, Portugal

**Keywords:** neurological disorders, movement quantification, inertial sensors, smart textiles

## Abstract

The development of wearable health systems has been the focus of many researchers who aim to find solutions in healthcare. Additionally, the large potential of textiles to integrate electronics, together with the comfort and usability they provide, has contributed to the development of smart garments in this area. In the field of neurological disorders with motor impairment, clinicians look for wearable devices that may provide quantification of movement symptoms. Neurological disorders affect different motion abilities thus requiring different needs in movement quantification. With this background we designed and developed an inertial textile-embedded wearable device that is adaptable to different movement-disorders quantification requirements. This adaptative device is composed of a low-power 9-axis inertial unit, a customised textile band and a web and Android cross application used for data collection, debug and calibration. The textile band comprises a snap buttons system that allows the attachment of the inertial unit, as well as its connection with the analog sensors through conductive textile. The resulting system is easily adaptable for quantification of multiple motor symptoms in different parts of the body, such as rigidity, tremor and bradykinesia assessments, gait analysis, among others. In our project, the system was applied for a specific use-case of wrist rigidity quantification during Deep Brain Stimulation surgeries, showing its high versatility and receiving very positive feedback from patients and doctors.

## 1. Introduction and Related Work

The advances in electronics and, in particular, in microelectronics, together with the emergence of new wireless communication protocols have been contributing to the successful development of wearable devices [1,2]. These can be defined as “devices that can be worn or mated with human skin, to continuously and closely monitor an individual’s activities, without interrupting or limiting the user’s motion” [3]. Wearable devices started to be used mainly for military and fitness purposes, however, with the recent focus on ageing population and the consequent will to ensure better health conditions to people in general, an effort have been made in both industry and research to design and develop wearable devices to be used in the healthcare environment [4].

Wearable Health Devices, as they are known, have been used for health and wellness monitoring, home rehabilitation, treatment efficiency assessment and early detection of disorders [1]. All these categories imply the use of wearable devices for monitoring human physiological signals, such as electrocardiogram, heart rate, blood pressure, respiratory rate, body temperature and motion [5]. Besides the physiological tracking, a wearable health device must transmit data in a reliable and secure way, demand low power, be as less invasive as possible, comfortable and very easy to use [5]. To ensure these three last requirements, engineers rely on the synergy between electronics and textiles, originating the concept of smart textiles. Smart textiles can be defined as “textile products such as fibers and filaments, yarns together with woven, knitted or non-woven structures, which can interact with the environment/user” [6]. Other than providing comfort and usability to wearable systems, smart textiles also have the potential to add flexibility, deformability and stretchable interconnections between electronic components [4].

In the scope of neurological disorders, wearable health devices have been widely used, instead of clinical rating scales, to quantify movement symptoms [7]. The non-linearity, the ceiling and floor effects and the inter-observation variability of clinical rating scales makes them highly subjective and with a low level of reliability [8]. For this reason, several research centers and companies have been recently working in body motion tracking with wearable devices of patients suffering from Parkinson’s Disease (PD) [9,10,11,12,13,14,15], Epilepsy [11,15,16], Stroke [11,17], Multiple Sclerosis [15,18], among others. However, these body motion tracking systems oriented-development for a specific disease or symptom(s) reveals limitation in their user, such as the ones discussed in the two next sub-sections.

The work presented in this paper focused on the development of a versatile textile embedded wearable device which provides inertial and analog raw data that can be used for the quantification of neurological motor symptoms, such as rigidity, tremor, bradykinesia and gait analysis. As example, rigidity quantification is achieved in Reference [19] by collecting angular velocity data from the wrist, tremor and bradykinesia are quantified in References [9] and [14], respectively, through the acquisition of accelerometer and gyroscope data from the finger. Gait analysis is performed by obtaining and processing accelerometer, gyroscope and analog data from the ankle [20]. Therefore, it was important to first understand the common needs in this field and to be aware of the limitations, discussed below, of recent research and industrial projects that aim to support neurological diseases. This helped in the development of the innovative concept conducted in this work, that is, an innovative wearable system for movement quantification that fulfills important requirements such as be comfortable, user-friendly, low power, broadcast viable and validated data and be versatile to be adapted for different use-cases.

With the purpose of quantifying motor symptoms using wearable devices in the area of neurological diseases, developers must make several technical decisions about their systems. Considering their target patients, they must concern with the specificity and number of sensors to use, the kind of technology to implement (for data transference, visualization and processing), the system running time, the body part(s) to track and the comfort and usability from the users’ point of view. Among other facts, these decisions depend on the systems being meant to embody a scientific proof-of-concept or being consolidated to enter the market.

In the following sub-sections, several aspects of different wearable health devices, developed in both research and industry community, are discussed in two main segments. The first focuses on technical aspects of the devices, such as the aforementioned ones. The second conducts an analysis about the devices textile integration.

### 1.1. Devices Technical Aspects

Micro electromechanical systems inertial measurement units (MEMS IMUs), which are miniaturized components comprising a 3-axis accelerometer, gyroscope and often magnetometer, are the most implemented sensors for motion tracking [7,9,10,11,12,13,14,15,16,17,18,20]. They are preferred amongst developers for their small size, light weight and high precision. However, depending on the aim of the project, the use of additional sensors, such as electromyographic [15,17], force [17,20] and flex [13,20] sensors are also common. These provide more detailed information about the muscles’ activity, the performed force and the joints angles, respectively.

To process and visualize sensitive data, developers use either a computer [9,11,12,13,14,17], a smartphone [7] or both [10,15,16,18,20]. At first sight, the single use of computers seems to be less practical, since smartphones are smaller, easier to use and carry and usually have enough processing capabilities. However, in cases where the intention is not to process data in real time [9,12,13,14,17], computers are a better option for data management and offline processing, due to their larger memory and better processing capabilities. The use of smartphones is more viable when the project goal is to visualize data and its processing result in real time [7]. Some research groups opt for implementing both external devices in their systems, taking advantage of computers better processing capabilities and the usability of smartphones to visualize data and results [18,20].

It is important to note that, despite the aforementioned research projects being focused on both home and hospital monitoring, neither of them has specific restrictions in relation to the electronic devices to use and the systems sizes. If more restrictions were imposed, for example in surgical environments, where the conditions of hygiene, space and stress are demanding, these systems would need to be as small and as simple as possible.

**Figure 1:**
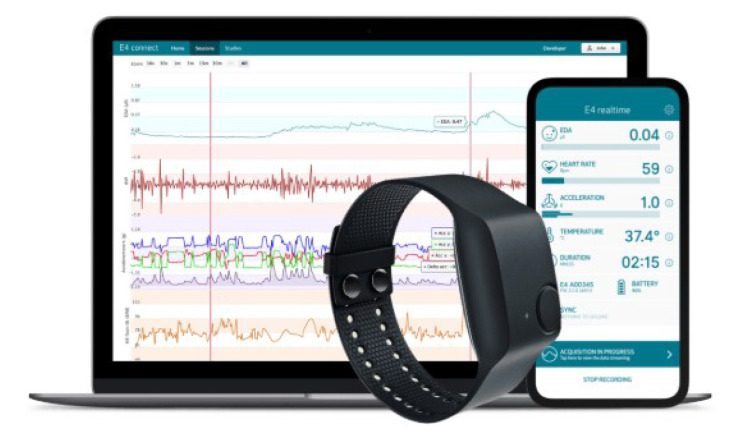
Empatica E4 Ecosystem implementing both computer (therapy management by the clinicians) and smartphone (data processing and display during patient use), retrieved from Reference [16].

Industrial projects (Figure 1) usually integrate smartphones and computers in their systems, mainly because smartphones are responsible for data visualization and processing, while computers are implemented for results monitoring and therapy management by clinicians [15,16].

In the context of data transference between the sensing systems and the external devices, a concern in applying wireless protocols is noted. Among different wireless options, authors often use Bluetooth [9,17], Bluetooth Low Energy (BLE) [7,11,13,15,16,18,20] or Wi-Fi [10,12] to broadcast data for further processing. Wi-fi is usually implemented in cases of long-distance communication or when a large amount of data has to be exchanged [10]. Comparing Bluetooth with BLE, which are used for short-distance communication, the BLE protocol is preferred since it provides a more efficient energy management [21].

The use of wireless protocols in projects of motion tracking is a wise choice, since the existence of wires can easily restrict the users’ movement. The project described in Reference [13], where the goal is to quantify bradykinesia, is an exception to the implementation of wireless protocols. In this project, authors established an USB connection to the computer where data are visualized and processed. With the wire connection, the finger’s movement will be restricted and it can even bother the user, therefore wireless options are preferred.

Finally, regarding the devices autonomy, authors do not generally share details about the process of choosing the battery for their systems but they usually mention the autonomy time of the wearable devices and the batteries capacities. With this information and based on the research projects mentioned in this paper [7,9,10,11,12,13,14,17,18,20], it was possible to conclude that large capacity batteries, in the range of 430–1800 mAh, are typically used, allowing autonomy timeframes of 12 to 24 h. Despite the autonomy timeframe being acceptable for the authors, the use of large capacity batteries also means that larger batteries are used, resulting in large and heavy systems. This may not be a problem for research projects where proof-of-concepts are being developed but the same is not applicable to market-oriented projects. In fact, as expected, market-oriented projects show more efficiency in this field. In Reference [15], 36 h of autonomy is achieved with a 15 mAh rechargeable battery and in Reference [16] a 260 mAh battery is used to provide an autonomy of 24 h.

The choice of batteries highly depends on several aspects that can change from project to project. Examples are the amount and nature of the sensors and the real-time or offline data processing. Therefore, what must be retrieved from this analysis is that it is of utmost importance for authors, in accordance to their systems and goals, to apply low power technologies in order to achieve smaller and lighter products.

### 1.2. Devices Textile Integration

**Figure 2:**
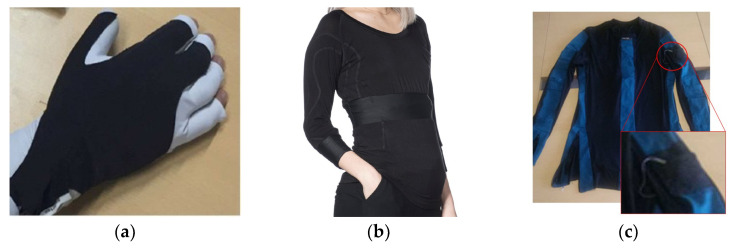
Different textile pieces integrating electronics for movement quantification in different areas of the body: (**a**) Glove for recognition of eight fundamental hand positions for Stroke patients [17]; (**b**) T-shirt to quantify symptoms of Parkinson’s Disease (PD), Epilepsy and Stroke patients [11]. (**c**) T-shirt to assess Stroke patients arm movement during daily activities [17].

Besides the technical details, aesthetic, usability and comfort are also essential requirements for a wearable health device. To meet these requirements, researchers often develop smart textiles in scientific projects where additional sensors are implemented [11,13,17,20] or in projects where the aim is to track motion of a large part of the body, such as the trunk [7]. Generally, they use already existent pieces of clothing, such as socks [20], sport gloves [12,13] (Figure 2a) and swimsuits [7] or they produce new ones, for example. t-shirts (Figure 2b,c), trousers and gloves [11,17].

The use of pieces of clothing may not be that practical for both, developers, who must produce different sizes of those pieces and users, who frequently have difficulties in getting dressed. On the opposite side, small customised pieces adaptable to different parts of the body would be more appropriate for people with neurological diseases. This would facilitate the process of putting on and removing the devices, which could be sized independently.

As noted in the aforementioned research projects, inertial sensors are usually integrated in textiles through sewn pockets [11,17] (Figure 2c) or glued with a specific adhesive [7]. The integration of additional sensors is made through hand stitching [13,20], which is a thorough task that can become an obstacle to the system aesthetic and its potential mass production. Alternatively, they can be sewn with a machine [11,17] (Figure 2c), which is a task that must also be carried carefully due to the risk of harming the sensors [13]. The communication between the multiple sensors is sometimes made through cables, that can be hided in channels [11,17] (Figure 2c) or exposed [20].

Nevertheless, the use of cables is not a viable option for motion tracking systems. Cables stretch during the movement may interfere with the users’ performance and/or cause the cables to be released. Thus, as an alternative, some researchers have implemented conductive fabrics to connect different sensors (see glove on Figure 2a). These are mainly used when additional textile sensors are implemented, such as electrodes (ECG [11] or EMG [17]) and flex sensors [13].

In systems where electronics are not integrated in textiles, researchers attach the components to the users’ bodies resorting to standard elastic bandages with Velcro applications [10,12,14] or other standard accessories [18].

Based on our research, in industrial projects, smart textiles are not usually implemented for motion tracking of neurological patients. Instead, these projects use materials like silicone, in form of wrist bands [16] (Figure 1) or other materials [15] to integrate their electronic components. When textiles are used in industrial projects, electronic is generally inside rigid boxes which are then attached to the textiles (Embrace Watch [16]; MVN Analyze Model [22]).

As seen in the revision of the state of art, the scientific community is making efforts to develop textile embedded wearable inertial systems for motion tracking of neurological patients. Though it is still noted a lack of a small, light and low power inertial device, that can be easily integrated in a textile piece, adaptable to different parts of the body. Market-oriented projects advance at a high speed but they do not seem to be exploring the potential of textiles to incorporate electronics. Therefore, a comfortable (to wear) and easy to use motion tracking system that can rapidly be adapted to different environments (home, hospital, operating room) and different parts of the body, integrating every day materials (textiles), would be a noticeable advance in this field.

### 1.3. SnapKi Development Goals

In this paper, we present the SnapKi—an innovative textile embedded inertial wearable device which can be used for the quantification of different symptoms in different parts of the body. The SnapKi is attached to a seamless, comfortable and unique size textile piece that supports the hardware through a snap buttons system. In addition, a web cross application was developed for real-time data visualization and saving in a CSV format.

The SnapKi includes a 9 DoF IMU (3-axis accelerometer, gyroscope and magnetometer), a BLE module with a microcontroller unit (MCU) integrated and a small battery. During the components’ procurement, our priority was to choose the most cutting-edge and miniaturized technology, in order to obtain the smallest and lowest power device. Electronics is properly coated with a resistant textile material that protects it from possible damages. Furthermore, the device contains a snap buttons system that enables its connection to two analog sensors and to the textile piece. These analog sensors can be of any type, such as force, flex, pressure, temperature, light or sound and they can be made of any materials, textile or others since it is possible to design a simple connection to the snap buttons. The snap buttons also allow an easy placement and removal of the hardware, providing the possibility of washing the textile.

This system easily adapts to different use cases, being necessary intervention only in two particular fields—setup adaptation and data acquisition features adaptation. Regarding the setup adaptation, it is only required a few modifications on the textile piece—its shape and size can be readjusted according to the part of the body where it is aimed to track motion. The data acquisition features adaptation consists in the adjustment of settings like sensors measurement frequency, inertial sensors full scale and analog sensors sensitivity. These settings can be adjusted through the developed web platform, which is also very useful for debugging and sensors calibration. In this way, we are able to rapidly ensure measurement precision according to the use case.

In order to test this adaptative wearable device in a specific use-case, this was applied in the iHandU system, which is a peculiar system for wrist rigidity quantitative assessment during Deep Brain Stimulation (DBS) surgeries. In specific moments of the surgery, the textile band with the hardware unit integrated is placed in the patient’s hand palm while the clinician performs the wrist rigidity evaluation movement. As analog sensors, customized force sensors that measure the force the clinician performs on the patient hand were developed, since this force can interfere in the rigidity quantification algorithm. Angular velocity data is transmitted to a smartphone, where it is processed and a score for the patient’s rigidity improvement, under certain brain stimulation parameters, is computed [19]. We were able to obtain very positive feedback on the SnapKi integration in the iHandU system. In fact, interviews were made to four neurosurgeons who were present in 5 Deep Brain Stimulation surgeries where the SnapKi device was used for wrist rigidity evaluations. The doctors were pleased with the fact that the current version of the textile band was thinner and more stretchable and the hardware inertial unit was smaller than the previous version [18]. These features would make the wearable device more comfortable, easy-to-use and able to fit different hand sizes. They also stated the absence of common allergies or red stains appearing on the patients’ hands in these 5 cases. Lastly, they claimed that the assessment process was easier and that they felt it was more reliable due to the strong attachment of the textile to the patient hand.

This paper is divided in four main sections. In Section 2, the SnapKi wearable health device and its surrounding system is presented, that is, all details regarding hardware and firmware development, textile adaptation and sensors calibration and validation are explained. Section 3 introduces the adaptation of the SnapKi to the iHandU system. Here, the modifications of the textile band and the production of the force sensors are described. Furthermore, the results obtained from a comparison between the previous iHandU system hardware and the SnapKi are presented. Section 4 illustrates the conclusions to retrieve from this project and some future directions.

## 2. SnapKi: New Easy-to-Adapt Wearable Device for Movement Quantification

**Figure 3:**
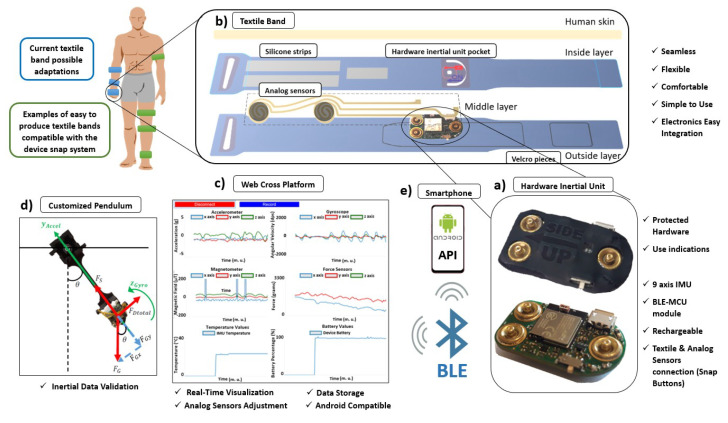
SnapKi wearable device surrounding system: (**a**) SnapKi hardware inertial unit exposed and coated. (**b**) Customized textile band for hardware inertial unit and analog sensors integration. (**c**) Web cross platform for data visualization and saving. (**d**) Customized pendulum for inertial data validation & device calibration. (**e**) Smartphone for data processing and visualization during patients use.

SnapKi is a wearable health device designed for movement symptoms quantification of neurological patients. It complies with all the requirements to be used in home, clinical and surgical environments and it relies on an innovative concept that allows it to be easily adapted and reconfigured (Figure 3) to different parts of the patient’s body. The device consists of a hardware inertial unit (Figure 3a) with the possibility to connect to two analog sensors (example of textile force sensors for a specific use case explained in Section 3), that fits in a customizable textile band (Figure 3b). This textile band can be rapidly modified and adapted to different purposes, that is, quantification of different symptoms in different parts of the body.

The device data is transmitted through BLE to a computer (Figure 3c) or to a smartphone (Figure 3e). In clinical or home scenarios, the smartphone is used during the users’ movement symptoms quantitative evaluation for data processing and results display. To support the use of the Android smartphone an Application Programming Interface (API) was created to easily manage received data from the inertial unit. On the other hand, the computer can be used in lab environment for raw data visualization and testing, sensors reconfiguration and storage using a web cross application specifically designed for this purpose (Figure 3c). Finally, a customized pendulum (Figure 3d) was created to perform validation tests on the SnapKi before it gets to the user, ensuring inertial data reliability.

### 2.1. Hardware Inertial Unit

**Figure 4:**
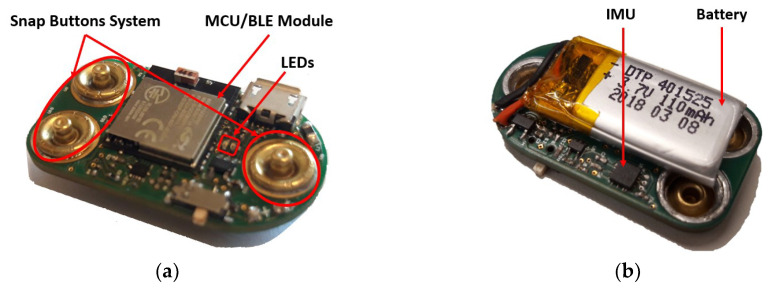
Hardware inertial unit: (**a**) Top view with the microcontroller unit (MCU)/ Bluetooth Low Energy (BLE) module, snap buttons system, on/off button and USB entry for device charging; (**b**) Bottom view with a small battery and the Inertial Measurement Unit.

The inertial sensor implemented in the hardware unit (Figure 4) is the ICM-20948. This is a 9 axis IMU and its excellent price-size relation was the main reason of our choice (Figure 4b). The accelerometer, gyroscope and magnetometer were set with a full scale of 4 g, 1000 dps and 4800 µT, respectively. In addition, they were set to make measurements at approximately 50 Hz. These parameters, that is, the inertial sensors measurement frequency and scales, can be modified through the web cross application, according to the purpose of the project where the device is applied.

The communication between the IMU and the MCU is made through the Inter-Integrated Circuit (I^2^C) protocol because it only requires two lines for data transference, reducing the space needed for its implementation what is an advantage in small hardware designs.

The MCU is integrated in a BLE module (BGM111), which is responsible not only for managing and processing data but also transmitting it to an external device (Figure 4a). Combining the two largest components in a unique packaging, BGM111 allows to save space in the hardware without compromising the MCU or the BLE efficiency. Besides the inertial data, the hardware is also able to acquire information from two external analog sensors.

The analog sensors connect to the device through three snap buttons (Figure 4a), where two of them are responsible for conducting the sensitive signals and the third one is the feeding line (VCC line). These lines are then linked with the BGM111 ADC where they are converted to digital signals and sampled at a 50 Hz sample rate. As complementary information, the IMU temperature and the device battery percentage are being read by the MCU at 0.5 Hz.

Finally, two LEDs were implemented in the hardware (blue and red) with the purpose of having visual indicators of the device status. The blue one flashes while the device is transmitting and it flashes at a higher frequency when the battery is under 20%. The red one turns on while the device is charging and off when it is fully charged.

#### 2.1.1. Device Energy Consumption

To ensure the maximum energy efficiency of the device, during the components’ procurement, efforts were made to choose low power components. In accordance with these efforts, we opted by using BLE to exchange data between the wearable and an external device and a low-power 9 axis IMU to acquire the data.

To study and optimize the device energy consumption, we made an energy consumption analysis to find out how to exchange data with the lowest power consumption. This analysis was conducted using the Energy Profiler tool of the Simplicity Studio IDE, where the firmware was developed (https://www.silabs.com/products/development-tools/software/simplicity-studio) [23]. The main requirement was that at least one sample of each sensor (accelerometer, gyroscope, magnetometer and two analog) had to be sent to the external device at the same time. Having this into account, two data transmission features were considered:

1. Notifications number—the objective was to conclude if it would be more power efficient to separate data in different characteristics, resulting in a simultaneous transmission of multiple smaller notifications or if it would be more power efficient to send a single large notification.
2. Notifications Size—having defined the number of notifications, the aim was to understand how much data they could contain, knowing that larger data notifications would be sent with less frequency.

Considering the main requirement, one sample of each sensor was used to perform the tests of the number of notifications. Data was grouped in 4 different ways and the Energy Profiler was used to infer the estimated current consumption in each scenario, as presented in Table 1.

With these results, we concluded that, it would be more efficient for the system to send one sample of each sensor in one single notification.

Having this defined, the tests of the notifications size were performed. The intention was to confirm how big the notification could be, that is, how many samples per sensor could be sent to an external device within the same notification and therefore at the same instant. The maximum number of samples per sensor tested was five, since the GATT protocol [24] establishes a maximum number of bytes to be transmitted in a single notification of 255 (six samples per sensor would result in more than 255 bytes). Results of the second data transmission feature tests are presented in Table 2.

It was concluded that sending 5 samples from each sensor in a single notification at each 100 ms would provide a maximum efficiency of the system energy. Regarding the complementary information (temperature and battery percentage), it is being sent at a lower rate (0.5 Hz), therefore it does not interfere with the average consumption of the device.

With this implementation, while transmitting, the device consumes, in average, 9 mA. Using a 110 mAh capacity battery it achieves 13 h of autonomy.

#### 2.1.2. Hardware Format & Protection

One of the main priorities of this project was to develop the smallest hardware possible. In fact, after the efforts made in the components procurement to choose small size components and in managing their location in the device, a very small and light hardware, in comparison with the state of art devices (Table 3), was achieved—20.45 × 37.19 × 5 mm and 7 g weight (snap buttons already soldered). 

**Figure 5:**
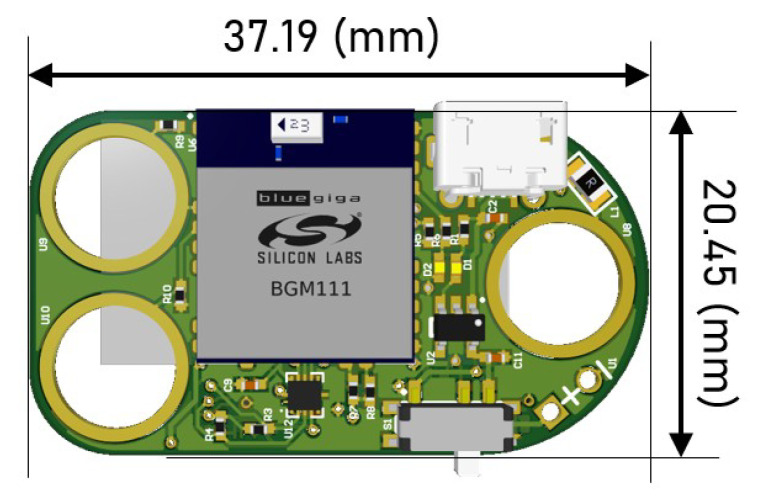
Hardware inertial unit design in a CAD software (dimensions explicit).

Regarding the hardware format, it was important to create a board with rounded edges to avoid causing any discomfort to the user (Figure 5).

**Figure 6:**
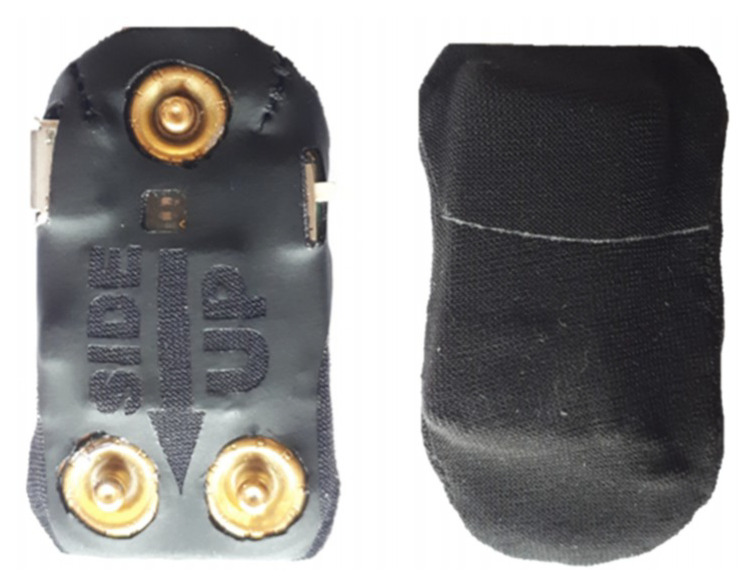
Hardware inertial unit coated with a slim, resistant textile.

To protect the hardware, a resistant textile that perfectly adjusts to the device was used (Figure 6). This textile was developed in partnership with Petratex Confecções, S.A. company and it allows us to effectively protect the electronics without using the typical boxes, which are stiff and often increase the wearable size [10,12,14]. This protective textile has openings to enable the connection of the snap buttons, the visualization of the LEDs lights, the access to the on/off button and the device plug. Finally, it has a label indicator for the user to know how to insert the device in the textile piece.

### 2.2. Textile Band

A textile band was created, in partnership with Petratex Confecções, S.A. company, to incorporate the SnapKi and allow its use in different parts of the body. This textile is washable and re-usable, including a label with the washing conditions to ensure its best preservation.

**Figure 7:**
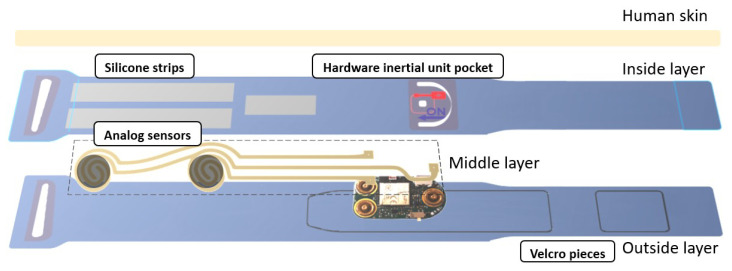
Customized textile band for sensors integration and attachment to the human body.

The textile band (Figure 7) is constituted by three overlapped layers of hypoallergenic (inside layer), stretchable (all layers) and comfortable textiles (all layers). These are glued together to prevent the existence of seams, which can bother the user.

In the inside layer, that is, the layer in contact with the skin, the hardware inertial unit is inserted in a slim pocket, which comprises the female parts of the snap buttons, strengthening the device fixing. Through the snap buttons, a solid connection of the device to the textile is established, the device placing and removal is easier and, because they are made of a conductive material, the device connection with analog sensors is possible. This connection is made through a conductive silver coated textile, which is in the middle layer of the textile piece. The hardware inertial unit pocket allows the visibility of the on/off button, the USB entry and the LEDs light, through a window coated with transparent film. Furthermore, it has labeled indications for the user to know how to manage these last functions. Still in the inside layer, three strips of silicone prevent the band from sliding.

The band closes around the users’ body thanks to a buckle and two pieces of Velcro, which are in the outside layer.

To note that all these features of the band are easily modified in order to adapt it for different parts of the body. For example, in a gait analysis project, the band would be placed in the ankle, as represented in Figure 3 (in green) and therefore it would have a longer length. Furthermore, the band shape would be adapted to go around the ankle, avoiding bothering the patient. Other example of the band adaptation for the hand (Figure 3 in blue) is given in Section 3.

### 2.3. Web Cross Application 

Visualizing the data transmitted by the device is of utmost importance for debugging and calibration purposes during the development of the wearable device. Therefore, a web cross application was created, based on JavaScript and HTML. This web application allows a real-time visualization of the device raw data for testing before its use on real-life studies. Moreover, it also allows to store the acquired data in a CSV format file for preliminary tests and data analysis. Methods of the Web Bluetooth API from Google were used to connect the BLE device with the Chrome browser. Since the SnapKi can be connected with different analog sensors, different units and ranges of values, a code framework that can be easily editable was created to integrate new information. Therefore, the code is ready to rapidly integrate data from new sensors, which is very useful for the textile band adaptation to other parts of the body, where different sensors may be needed. In fact, the validation and calibration of the device data using this platform is an indispensable step in the adaptation of the SnapKi to different systems.

**Figure 8:**
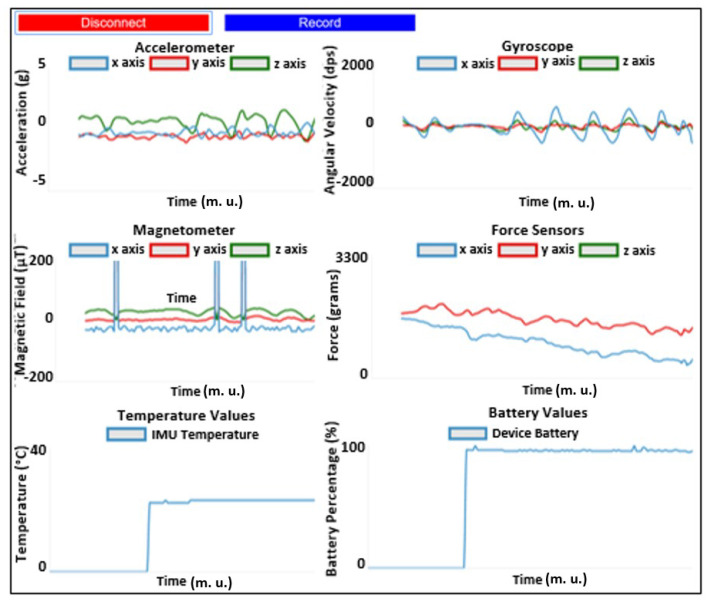
Chrome JavaScript web application for real-time data visualization and saving. Graphics enumeration from top to bottom and left to right: accelerometer, gyroscope, magnetometer, both analog sensors (force sensors used for this example), temperature and device battery percentage.

This intuitive and user-friendly web page (Figure 8) comprises two main buttons, a “Connect/Disconnect” button to start/stop receiving data from the device and a “Record/Stop & Save” button to start/stop recording data and proceed to its saving. Data is displayed in six graphs—one for each of the four sensors implemented in the device and two more to display temperature and battery percentage information.

### 2.4. Sensors Data Validation

Having completed the development of the system hardware and firmware, the intention was to validate the inertial data it provides. This is an imperative step to have confidence in the collected data in order to use it for medical purposes. To accomplish this, a pendulum system was built. The theoretical movement data of this pendulum can be computationally obtained and then compared with the experimental one in order to validate the raw data of the SnapKi inertial Unit.

**Figure 9:**
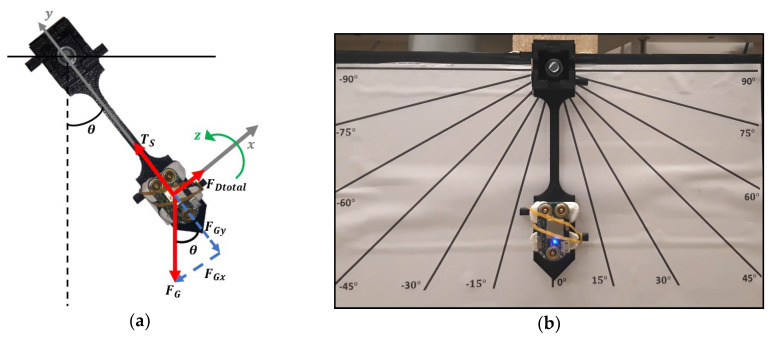
Customized pendulum for inertial data validation: (**a**) Forces diagram of the pendulum system; (**b**) Pendulum with the hardware attached and the marked angles behind for data acquisition.

The pendulum itself consists in a 3D piece designed in a CAD software (Figure 9a). It has a hole on the top which is useful for the pendulum rotation and attachment to the wooden board that supports it. For this purpose, the external ring of a bearing was first glued to the pendulum support. Then, the pendulum was attached to the internal ring of the bearing by crossing a screw, two washers and two nuts through the pendulum hole. In this way, the pendulum was properly attached to the support and therefore able to rotate. On the bottom tip of the pendulum, the device can be assembled in a groove and held through elastics that fit on two lateral hooks. Furthermore, a cardboard with the angles marked in relation to pendulum rest position (vertical direction) was added to the system. As a result, we achieved a pendulum (Figure 9b) with 10.5 cm length, 23 g of bob mass (device included). According to the pendulum features, the damping coefficient was experimentally estimated, in MATLAB^®^ (The MathWorks, Inc., Natick, MA, USA), based on Equation (1) [25]:

(1) $$\ddot{\theta }=-\left(\frac{g}{L}\right)\sin\theta -K^{\prime }\dot{\theta },$$

where $\ddot{\theta }$ is the angular acceleration of the bob, $\dot{\theta }$ is its angular velocity, θ is its angular position, g is the gravity constant, L is the pendulum length and finally, $K^{\prime }$ is the ratio between the damping coefficient of the system and the bob mass. The damping coefficient algorithm consists in the computation of the oscillation differences between one experimental curve and several theoretical curves achieved with different damping coefficients. With this comparison was aimed to understand the damping coefficient that better approximates the curves in terms of oscillation timings. This procedure was repeated for 100 experimental linear acceleration curves and 100 experimental angular velocity curves. In the end, the damping coefficients obtained from the 200 experiments comparison were averaged, resulting in a $K^{\prime }$ = 0.7.

The next step was to use the customized pendulum to validate linear acceleration and angular velocity data collected by the device.

First, considering the pendulum characteristics, the physical equation of a damped pendulum system (equation 1) was used to estimate the angular velocity theoretical movement curve, associated to the pendulum. This equation was solved, for the z direction, using the Runge-Kutta 4th order method [26] in MATLAB^®^ (The MathWorks, Inc., Natick, MA, USA), with the following initial conditions—angular position −90°; angular velocity −0 dps.

To achieve linear acceleration, the 2nd Newton’s law was used for the forces acting on the pendulum in the y direction, obtaining the following equation:

(2) $$a_{y}=L{\dot{\theta }}^{2}+g\cos\theta ,$$

where $a_{y}$ is the linear acceleration of the pendulum in the y axis.

Then, the device was submitted to the pendulum movement under the same initial conditions. Through the developed web platform, the linear acceleration and angular velocity data acquired by the device were recorded and saved from the beginning of the movement until it stops by itself. The experimental data was obtained by repeating this procedure 50 times, after ensuring that this amount of data would be enough to give statistic confidence [27].

**Figure 10:**
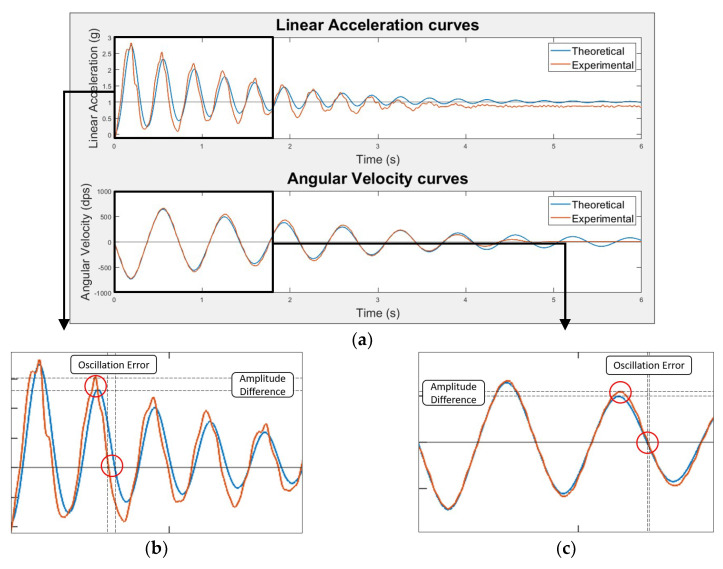
Inertial data validation results: (**a**) Example of a theoretical and experimental angular velocity and linear acceleration signal; (**b**) Linear acceleration curve corresponding to pendulum first 5 oscillations with the comparison results highlighted; (**c**) Angular velocity curve corresponding to pendulum first 5 oscillations with the comparison results highlighted.

After obtaining both the theoretical and experimental data, their comparison was conducted (Figure 10a). To compare the experimental curves with the theoretical ones, a MATLAB^®^ (The MathWorks, Inc., Natick, MA, USA), algorithm, which considered only the first five oscillations of the pendulum, where the experimental curves could be approximated to the theoretical ones, was developed. The comparison with the theoretical curves was conducted for each of the 50 experiments and they were based on two factors:

- Mean Oscillation Error (OE)—averaged percentage difference of the intersections with the curves point of balance (y = 1 g for linear acceleration and y = 0 dps for angular velocity) in between the theoretical and experimental curves.
- Lin’s Concordant Correlation Coefficient (CCC)—reflects how well a new measurement (experimental) reproduces a gold standard one (theoretical). Perfect agreement—Lin’s CCC = 1 [28].

These are depicted for linear acceleration (Figure 10b) and angular velocity (Figure 10c) curves.

The comparison factors obtained for each of the 50 experiments were then averaged and are depicted in Table 4.

These results express low errors in terms of oscillation timings and amplitudes disparities for linear acceleration and angular velocity data. In fact, the similarity between the experimental and theoretical data is visible in Figure 10. Consequently, we can successfully validate and have confidence in the inertial data provided by the device.

The experimental linear acceleration data presents a time desynchronization around the 4% in relation to the theoretical data and a reproducibility, in amplitude, of 0.92. This slightly deviation can be explained by the fact that linear acceleration values in the different directions of the accelerometer are dependent on its orientation (gravity effect). In other words, if the device is not perfectly aligned with the pendulum vertical direction, the linear acceleration weights in the three axes of the accelerometer will be different than the expected. In a perfect scenario, when stopped, the linear acceleration value in the y direction of the accelerometer must be 1 g, which is not verified in Figure 10a.

Considering the angular velocity data, we were pleased with the good results obtained.

Note that the validation was made to one axis of each sensor, because it was assumed that the others reveal a similar behavior.

Magnetometer data was not part of our priorities, since it is less used in the scope of neurological patients’ movement quantification [29]. Therefore, the magnetic field information collected by the SnapKi will be explored in the future.

## 3. Use Case: The Wearable Device in the iHandU System

**Figure 11:**
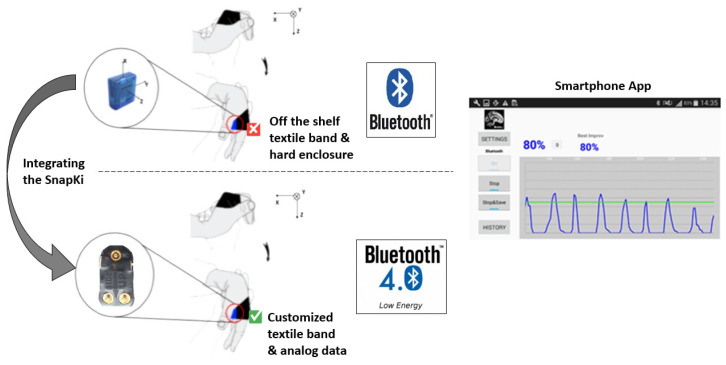
iHandU system architecture before and after the application of the of the SnapKi system. Differences: hardware inertial unit, textile band and data transference protocol (Bluetooth to BLE).

The iHandU system (Figure 11) started its development in 2015 [30,31] with the purpose of overcoming the subjectivity of the wrist rigidity improvement assessment of PD patients during DBS surgeries.

DBS surgeries consist in the implantation of electrodes in specific sites of the brain, where the electrical stimulation is able to alleviate motor symptoms. To set the stimulation parameters, during the surgery, doctors often evaluate the patients’ wrist rigidity. However, their evaluations are based on subjective scales, which bring problems such as lack of reliability, inter-observation variability, non-linearity, among others. As an answer to these issues, the iHandU system makes use of an inertial device, inserted in a band and a smartphone to provide the rigidity quantitative evaluation based on a patented method [32], supporting doctors in setting brain stimulation parameters. The inertial device transmits inertial data through Bluetooth to the smartphone, where it is processed and the rigidity improvement score is computed.

Since 2015, the system has been used weekly in surgeries at *Hospital de São João* (Porto, Portugal), under ethical consent and therefore, it went through several upgrades, according to patients and doctors’ feedback [19]. Until now, the upgrades were mainly focused on the rigidity quantification algorithm, being the hardware a piece of the system that was outdated—the inertial device was too large; low power protocols were not implemented; the device was inserted in an off-the-shelf and uncomfortable case and textile band; force sensors were not implemented to study the influence of the force doctors performed in the patients’ hand during the wrist rigidity movement evaluation.

The textile band and web application presented in this paper (Section 2) allowed the easy integration of the SnapKi device in the iHandU system, improving it to its best potential.

Believing in the iHandU system success with the SnapKi integrated, a startup, InSignals Neurotech, aims to take it to the international market reaching clinics and hospitals. In fact, the feedback from both patients and doctors who used the new iHandU system have been truly positive.

### 3.1. SnapKi Wearable Device Adaptation

With the SnapKi wearable device, the iHandU system comprises a smaller, lighter and low power hardware unit, which is inserted in a customized and comfortable textile band. Furthermore, the analog sensors included in the band were designed to allow the collection of force measurements (textile force sensors), overcoming one of the flaws of the iHandU system. 

For wrist rigidity assessment, the inertial device must be placed in the palm of the patients’ hand. In addition, to measure the force doctors perform in the patients’ back of the hand, force sensors are needed. To fulfil these needs, the textile band was rapidly adapted becoming suitable for the iHandU system. Furthermore, analog textile force sensors were developed using conductive textile and connected to the snap buttons system (analog entries) of the hardware inertial unit. These sensors were calibrated using the developed web application, which was modified to read their information. The textile developments, in both textile band and force sensors, were made in partnership with Petratex Confecções, S.A. company.

#### 3.1.1. Textile Band

**Figure 12:**
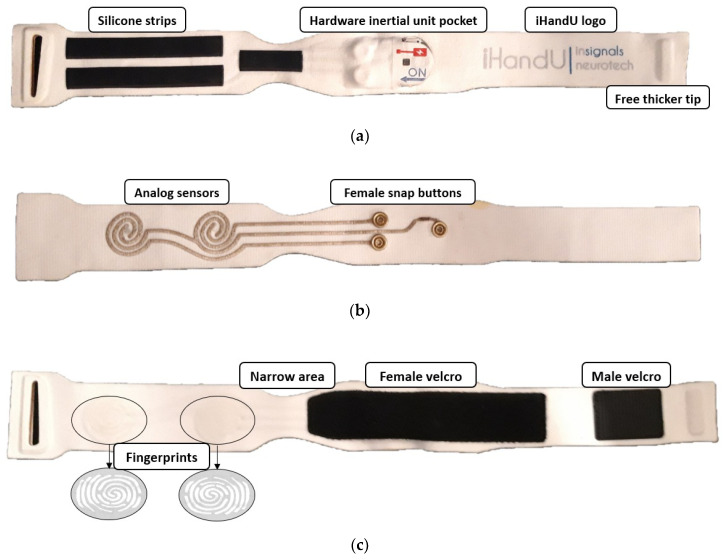
Textile band adaptation with new details to be suitable for the iHandU system. (**a**) Inside layer; (**b**) Middle layer; (**c**) Outside layer.

With regard to the band shape, a narrow area in the center was created (Figure 12). When the band is inserted in the hand, the narrow area must be placed between the thumb and index finger, removing any discomfort the patient might feel in this area.

Furthermore, two silicon fingerprints were placed in the outside layer of the band to help doctors identify where the force sensors are located (Figure 12c). This will help the clinicians to know where they should grab the hand of the patient correctly to perform their force in the area of the force sensors. These illustrative “instructions” can even contribute for a standard mode to grab the patient hand during wrist rigidity assessment, since it is still not coherent between clinicians.

**Figure 13:**
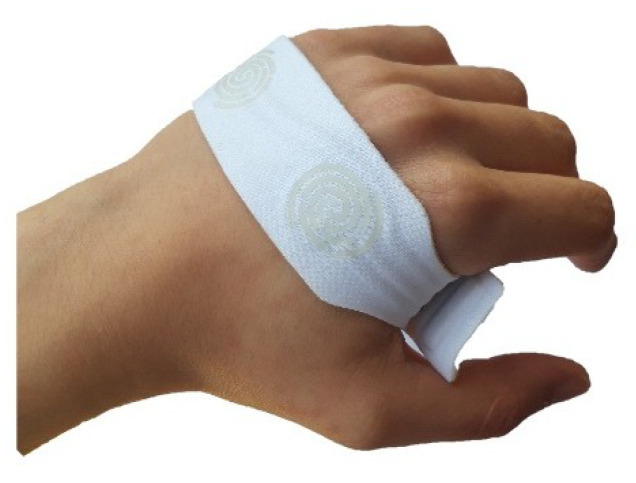
iHandU adapted textile band in closed mode.

Finally, the band is usually placed in the patient’s hand in its closed mode, that is, the free tip is inserted in the buckle, which joins the other tip through the pieces of male and female Velcro (Figure 12c). To maintain the band closed, a thicker strip on the free tip was implemented as a mechanism that offers some resistance to the band opening, allowing an easy and fast placement and removal of the band without its dismount. This because it is easier to insert the band in closed mode, that is, in a circular shape (Figure 13).

#### 3.1.2. Force Sensors

To measure the force performed by doctors on the patients’ hand, textile resistive force sensors were designed, produced and integrated in the middle layer of the textile band.

Resistive force sensors can be easily produced with two overlaid conductive materials, whose resistance changes when they are under pressure. The used materials were a silver coated textile, thanks to its good conduction and stretchable properties and a circular piece of Velostat. By choosing a stretchable conductive textile, the elasticity of the band is not compromised when it integrates the force sensors. Two strips of the conductive textile were cut and shaped intercalated in a spiral format (Figure 12b). One of the strips tip was connected to the VCC female snap button, while the other two were connected to the sensing female snap buttons. The piece of Velostat was then placed on the top of the conductive spiral. Therefore, when force is made on the Velostat, it gets in contact with the conductive textile. As a result, current starts to flow between the two conductive strips and the resistance decreases when force is applied. The resistance changing allows to pass more current between these two conductive strips and with a simple a voltage divider circuit, it is possible to read this resistance variation using the ADC. In this way, when the force sensors resistance changes, the circuit output also changes.

To calibrate the sensors, weights of known values were used. The goal was to adjust the voltage divider on-board resistor and therefore the sensitivity of the sensors to a range of values that matched the forces performed by doctors during the rigidity evaluations. Through tests in the lab, we concluded that the range of force the doctors make is 0–1.5 kg. With that being said, using different fixed resistors, four weights were placed on top of the sensors and their output voltage was visualized in the web platform. For each fixed resistor, exponential calibration lines were computed and the sensitive ranges were evaluated. From this, it was determined that the most appropriated fixed resistor for this purpose has a value of 5.1 kΩ (Table 5). The calibration line can be mathematically described by

(3) $$\mathrm{Force}\;(\mathrm{grams})=0.7145\;\cdot \;e^{0.0027\;\cdot \;Output\;Voltage\;\left(mV\right)},$$

with r^2^ = 0.9766.

### 3.2. SnapKi vs. Previous Hardware Inertial Data

**Figure 14:**
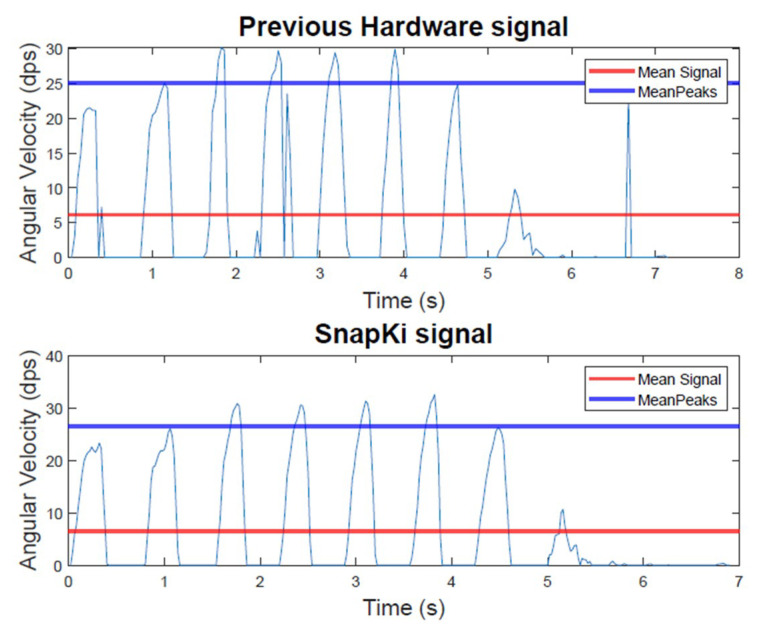
Previous iHandU hardware vs. SnapKi angular velocity signal obtained in a rigidity evaluation during Deep Brain Stimulation (DBS) surgery (mean signal and mean peaks highlighted).

The replacement of the previous iHandU system hardware by the SnapKi can only happen when the similarity of the angular velocity data provided by the two devices is ensured. Therefore, both devices have been used simultaneously in the rigidity evaluations during DBS surgeries. To be able to perform such synchronized data collection the Android API was incorporated in the iHandU system Mobile application. The acquired data was then organized in a dataset which, until now, accounts for 45 angular velocity signals collected during the patients’ wrist rigidity assessments, that is, passive flexion of the wrist, in 5 DBS surgeries. During the surgeries, both wrists of the 5 patients were assessed and, in average, 5 evaluations were recorded for each wrist, being that the stimulation parameters changed at each repetition. This process is ongoing, whereby more data will be collected. Then, all the signals obtained with both devices are compared in MATLAB^®^ (The MathWorks, Inc., Natick, MA, USA), which computes the difference between the signals mean and peaks and the similarity of their respective rigidity scores, in percentage. An example of the angular velocity signals obtained in a rigidity quantitative evaluation with the two devices is presented in Figure 14. Furthermore, the mean of the comparison results for the entire dataset is shown in Table 6.

From Figure 14 it is certain that the two signals are very similar. However, the results show a slightly difference between the angular velocity data obtained with the previous hardware and the SnapKi. This difference is related with the fact that the SnapKi is capable of obtaining detailed data. In addition, since the SnapKi acquires data at a higher frequency than the previous hardware, it is possible to conclude that it allows for the collection of more accurate data.

## 4. Conclusions & Future Work

In this project, a wearable health device capable of transmitting inertial and analog data through a low power protocol was achieved. This device has unique and versatile characteristics that enables it to easily adapt to different use-cases. With very few modifications, the textile piece enables the device placement in any part of the human body. Furthermore, the web cross application turns possible different sensitive data visualization, storing, testing and adjustment, allowing sensors further calibration and validation.

This work relied on the background accomplished during the previous years of using the iHandU system in surgical environments, on a weekly basis. This experience made us aware of the drawbacks that emerge in the quantitative measurement of neurological disorders patients’ movement in a generic way. Furthermore, we realized of the importance of providing a multi-purpose device capable of acquiring movement data in different scenarios and that always ensures the requirements of a comfortable and easy to use wearable device.

In fact, with the system presented in this paper, obstacles such as lack of comfort, difficulty in use and purpose-oriented systems are overcome. The SnapKi wearable device, besides being designed with special focus on user comfort, it also integrates hypoallergenic textile materials. Its mechanisms to put and remove the wearable were made easier and basic instructions are explicit in the textile band. Furthermore, the device is adaptable to quantify motor symptoms in different parts of the body. It can also measure different sensitive analog information, according to the use-case, without having to change the system all together. Finally, the development of the web application eased the tasks meant to be conducted in lab environment, such as the sensors calibration, validation and configuration adjustments to achieve more reliable data.

As future research, we aim to perform improvements related with the hardware, such as adding the possibility for the user to decide if they wish to acquire the analog data or not, even with the analog sensors being implemented. Additionally, we believe that the hardware size can be reduced, if the circuit work voltage decreases to 1.9 V (work voltage of the ICM-20948), thus eliminating the need of a voltage translator. Besides the hardware improvements, we also intend to explore other textile “snapable” bands for other use-cases that would be adaptable for other body parts, such as tights, knees, head, trunk, wrist, elbow.

## Figures and Tables

**Table 1:** Energy Consumption Analysis—Notification Number, the best result is highlighted.

Data Format	Notifications Number	Current while Transmitting (mA)
accel/gyro/mag/analog	4	9.86
(accel; gyro)/mag/analog	3	9.64
(accel; gyro)/(mag; analog)	2	9.40
(accel; gyro; mag; analog)	1	9.17

**Table 2:** Energy Consumption Analysis—Notifications Size, the best result is highlighted.

Samples from Each Sensor	Sending Period Interval (ms)	Current while Transmitting (mA)
1	20	9.17
2	40	9.08
3	60	9.47
4	80	9.08
5	100	9.07

**Table 3:** Dimension and weight of the devices cited in the state of art.

Reference	Dimension (mm)	Weight (grams)
[9]	41 × 48 × 17.8	31.6
[7]	36.6 (diameter); 10.6 (thickness)	30.0
[10]	52 × 37 × 13	26.3
[18]	55 × 20 × 15	-

**Table 4:** Results of the comparison factors between the theoretical and experimental linear acceleration and angular velocity curves (50 trials average).

Curve	Mean OE ± Std (n = 50)	Lin’s CCC ± Std (n = 50)
Linear Acceleration	4.07 ± 1.80%	0.92 ± 0.03
Angular Velocity	0.54 ± 0.27%	0.98 ± 0.01

**Table 5:** Analog force sensors output voltage (mV) in function of four known weights (grams). Values achieved with a fixed resistor of 5.1 kΩ.

Index	Weight (grams)	Sensor Output Voltage (mV)
W_1_	12	1000
W_2_	204	2200
W_3_	605	2519
W_4_	1196	2580

**Table 6:** Results of the comparison factors between the previous hardware and the SnapKi angular velocity signals (45 signals average).

Mean Comparison Factor (n = 45)	Value (%)
Difference of the signals mean	6
Difference of the signals peaks	4
Similarity of the rigidity scores	84
